# A search for quantitative trait loci controlling within-individual variation of physical activity traits in mice

**DOI:** 10.1186/1471-2156-11-83

**Published:** 2010-09-21

**Authors:** Larry J Leamy, Daniel Pomp, J Timothy Lightfoot

**Affiliations:** 1 Department of Biology, University of North Carolina at Charlotte, Charlotte, North Carolina 28223, USA; 2 Department of Genetics, University of North Carolina, Chapel Hill, NC 27599, USA; 3 Department of Nutrition, University of North Carolina, Chapel Hill, NC 27599, USA; 4 Department of Cell and Molecular Physiology, University of North Carolina, Chapel Hill, NC 27599, USA; 5 Carolina Center for Genome Science, University of North Carolina, Chapel Hill, NC 27599, USA; 6 Department of Health and Kinesiology, Texas A&M University, College Station, Texas 77845, USA

## Abstract

**Background:**

In recent years it has become increasingly apparent that physical inactivity can predispose individuals to a host of health problems. While many studies have analyzed the effect of various environmental factors on activity, we know much less about the genetic control of physical activity. Some studies in mice have discovered quantitative trait loci (QTL) influencing various physical activity traits, but mostly have analyzed inter-individual variation rather than variation in activity within individuals over time. We conducted a genome scan to identify QTLs controlling the distance, duration, and time run by mice over seven consecutive three-day intervals in an F_2 _population created by crossing two inbred strains (C57L/J and C3H/HeJ) that differed widely (average of nearly 300%) in their activity levels. Our objectives were (a) to see if we would find QTLs not originally discovered in a previous investigation that assessed these traits over the entire 21-day period and (b) to see if some of these QTLs discovered might affect the activity traits only in the early or in the late time intervals.

**Results:**

This analysis uncovered 39 different QTLs, over half of which were new. Some QTLs affected the activity traits only in the early time intervals and typically exhibited significant dominance effects whereas others affected activity only in the later age intervals and exhibited less dominance. We also analyzed the regression slopes of the activity traits over the intervals, and found several QTLs affecting these traits that generally mapped to unique genomic locations.

**Conclusions:**

It was concluded that the genetic architecture of physical activity in mice is much more complicated than has previously been recognized, and may change considerably depending on the age at which various activity measures are assessed.

## Background

Physical activity is an important behavior exhibited by most humans on a daily basis. Activity levels vary considerably among populations, sexes, and/or age cohorts [1,2], although in most populations, typically only a fraction of all individuals engage in vigorous or sustained exercise [3,4]. This is unfortunate since it has become increasingly apparent that physical inactivity can predispose individuals to a host of diseases such as heart failure and cancer [5-8]. Of particular recent concern are the sedentary life styles that are thought to promote obesity in increasing numbers of individuals [9].

Although there is a wealth of knowledge about the environmental factors that predispose individuals to physical inactivity, it is somewhat surprising that we still know comparatively little about the genetic control of voluntary exercise. It is clear that the heritability of various activity traits in humans is moderate to high in magnitude [9,10], although the identity of the genes responsible for this genetic variation remains largely unknown. Several recent studies in mice [11-19] have been successful in uncovering a number of QTLs for various wheel-running traits. These studies have given us information about the numbers and modes of action of the QTLs governing these traits, and thus have been useful in providing a preliminary view of the genetic control of physical activity in mice.

Nearly all of these studies have analyzed inter-individual variability, but it has been apparent for some time that physical activity levels vary within individuals over time as well [20,21]. A recent exception is a study conducted by Kelly *et al*. [19] who searched for QTLs affecting several wheel running traits in mice each day over a six day testing period. Interestingly, they discovered QTLs that commonly influenced activity over several days as well as other QTLs that affected activity measures in the mice only during the initial period (days 1 and 2) of testing [19]. This result has important implications for clinical therapies that seek to promote physical activity [22], because the development of these therapies will require an understanding of what genes are active at different times during the lifetime of individuals and how these genes function. However, more studies clearly are needed to discover to what extent there are QTLs governing physical activity traits only during specific intervals of time.

We previously conducted an interval mapping analysis of the average distance, duration, and speed run by mice during a three week (21 day) period, and were successful in discovering 4 significant QTLs (on chromosomes 9 and 13) and 14 suggestive QTLs that affected these traits, with many colocalizing to similar positions in the genome [12]. Since the three-week data were calculated from the average of daily measures, however, this population afforded an excellent opportunity to analyze these traits within individuals across several time intervals. We therefore pursued this by dividing the 21-day period into seven 3-day time intervals, and conducting a genome scan for QTLs affecting the three activity traits during each of these intervals.

We had two basic aims in our investigation: (1) We wanted to discover how many QTLs influence these traits over the different time intervals and identify which ones would correspond to those QTLs previously found for activity over the entire 21-day period. We expected to find novel QTLs for activity that we did not uncover in our original study. (2) We were particularly interested to see if we might find some QTLs affecting the traits only in the early, or only in the late, intervals, and if so, what pattern of effects they might exhibit.

## Methods

### The population and traits

We used an F_2 _population of mice produced from an original cross of two inbred strains, C57L/J and C3H/HeJ, because these strains exhibited a great deal of divergence in wheel-running activity levels [23]. Thus Lightfoot *et al*. [23] previously showed that the C57L/J mice ran approximately 390% further, 320% longer, and 176% faster than the C3H/HeJ mice. A total of 310 F_2 _mice were measured for three activity traits: total daily distance in kilometers and total daily exercise time in minutes (each was recorded every 24 hours), and average daily running speed in meters/minute obtained by dividing distance by duration. For all mice, this was accomplished with a solid surface running wheel mounted in their cages that interfaced with a computer that counted the total wheel revolutions and recorded the time each mouse spent exercising [12]. In all aspects of the rearing and consequent testing of these mice (see Lightfoot *et al*. [12,23] for details), we followed guidelines approved by the UNC Charlotte IACUC, the American Physiological Society, and the American College of Sports Medicine.

All mice were exposed to the running wheels when they were from 35 to 62 days of age and measured for distance, duration, and speed each day for several weeks. For the purposes of this study, however, we age-matched the available activity data to a 63-day-old start age to the extent possible. This resulted in a final sample of 297 mice that were 63 days of age and 13 mice that were 60 days of age at the start of testing. Prior to this start age, all 310 mice had previously been exposed to running wheels for an average of 13 days (range 1 to 28 days). Once testing started, the F_2 _mice were measured daily during a 21 day period for each of these three physical activity traits.

To analyze within-individual variation, we calculated the mean of the activity trait values for each successive three day interval. We found that single-day values were subject to considerable variation [4], and the distribution of these trait values typically tended to be non-normal. Averaged over three successive days, however, the activity traits exhibited normal distributions (*P *> 0.05) and less variation than single-day values. This averaging therefore resulted in values that were relatively more precise, thereby increasing the statistical power to detect QTLs influencing the activity traits. We also considered that seven timepoints were sufficient to ensure an adequate assessment of these traits over the 21-day period, and the phenotypic values of these traits in each of the seven intervals were used to provide snapshots in time as the mice aged throughout the 9- to 12-week period. Thus our basic intent was to assess the genetic changes that occurred during this 21-day time period. Distance, duration, and speed values over each of the 7 intervals are designated DT1-DT7, DR1-DR7, and SP1-SP7 throughout the analysis.

All mice were genotyped with the use of 129 single-nucleotide polymorphisms (SNPs) that differed between the C57L/J and C3H/HeJ progenitor strains. These SNPs provided coverage of all 20 chromosomes in the genome (1767 total cM) with an average intermarker interval of about 14 cM. A few SNPs were not resolved, so for the QTL analyses described below, sample sizes occasionally were reduced slightly from 310.

### Preliminary statistical analyses

Prior to the QTL analysis, we first tested the seven distance, duration, and speed traits for potential effects due to sex, litter size, rearing block, and age [12]. This was accomplished by using these factors in separate multivariate analyses of variance (MANOVAs) run for each of the activity traits. As expected given that nearly all of the mice were of the same age when testing started, age effects were not significant. However, all three other factors were found to be statistically significant in the MANOVAs (*P *< 0.01). All activity trait values therefore were adjusted for these effects by calculating residuals from the model and adding them to the appropriate means in the overall population.

We also tested for any potential effects of the number of days of exposure to the running wheel prior to the start of testing. This factor was not statistically significant for any of the activity traits throughout the age intervals, so did not require any further adjustments to the activity data. Because all mice had previously exercised and showed no effect due to the number of prior days of exposure, it should be borne in mind that any QTLs found for the activity traits in the first several intervals would not be expected to be those strictly for anxiety or other fear-related behavioral differences as was postulated for the mice in the study by Kelly *et al*. [19]. On the other hand, Milner and Crabbe [24] have shown that activity and anxiety behaviors are negatively related, so we cannot rule out the possibility that some of the QTLs found for the activity traits may also be those controlling some aspect of anxiety.

### QTL analyses

We used the regression approach of interval mapping [25] to search for QTLs influencing each of the 21 activity traits. We implemented this approach with canonical correlation analyses conducted at each location 2 cM apart on all 20 chromosomes, as previously explained in detail in Lightfoot *et al*. [12]. The analyses generated LPR values equivalent to LOD scores [26] and if they exceeded appropriate threshold values, were considered to indicate putative QTLs. Both chromosomewise and genomewise threshold values were determined by traditional permutation methods [27], with the shuffling of the phenotypic data among mice done by keeping all 21 activity values for each individual intact. Although chromosomewise threshold levels of significance are not as stringent as genomewise levels, their use increases the probability of detecting true QTLs. In addition, with a 5% type I error at the chromosomewise level for each of the 20 chromosomes, we should expect only one false positive result.

We also tested each chromosome for two-QTL and sex-specific QTL effects on the activity traits following procedures previously described [12]. Once the positions of putative QTLs were determined, we used multiple regression of the activity traits on the additive and dominance index values at each position to estimate additive (*a*) and dominance (*d*) genotypic values and the contribution (*r*^*2 *^values) for the QTLs.

We expected some of the QTLs to be commonly affecting the various activity traits throughout the time intervals. We therefore first inspected the positions of all QTLs found for the values of each of the activity traits throughout the time intervals to see if they colocalized. Where this occurred, we conducted pleiotropy tests using the procedure outlined by Knott and Haley [28]. This procedure generates a likelihood-ratio (chi-square) test statistic that if significant, suggested that the QTLs were in separate locations. A non-significant result, however, suggested that there could be a common QTL exerting pleiotropic effects on two or more of the activity traits. Where this occurred, we determined the best, single position (and confidence interval) for each pleiotropic QTL from an analysis combining all traits that the QTL affected.

As an alternate to the analysis of each of the activity traits over the seven intervals, for each mouse we used linear regression to calculate the slope of each of the three traits over all intervals. These slopes for distance, duration, and speed were composite traits that represented the changes in activity throughout the intervals, and we conducted QTL analyses on these traits in the same manner as already described. Any QTLs found for these slopes were assumed to indicate genomic regions influencing the trajectory of wheel-running activities across the entire 21-day period.

## Results

### Basic statistics

Basic statistics (means and standard deviations) for distance, duration, and speed throughout the seven time intervals are given in Additional file 1. As may be seen, the distance traveled by the mice averaged 5.76 to 6.50 km/day across these intervals, increasing for the first three intervals and then tending to decrease slightly or level off during the last four intervals. This same trend is seen for duration of time run (311.4 to 339.7 min/day) as well. Means for speed increase for the first 3 intervals, decrease some in the 4^th ^and 5^th ^interval, and then increase considerably in the last two intervals. Thus unlike distance or duration, the highest mean value for speed is reached in the last (7^th^) time interval. One-way analyses of variance showed that there were statistically significant (P < 0.01) overall differences among the 7 intervals for each of the three activity traits, and Tukey's post-hoc tests suggested that the individual pairwise differences were mostly between traits in intervals 1 and 2 versus those in later intervals. Standard deviations for each trait are comparable across the intervals.

For each of the three traits, Additional file 2 gives the pairwise correlations of values across the time intervals. All are positive in sign and statistically significant. Their magnitude is generally moderate to high, averaging 0.80 for the distance traits, 0.77 for the duration traits, and 0.75 for the speed traits. Correlations of values in closely-related intervals are higher than those in distant intervals. As an example, the correlations for distance in interval 1 with those for distance in each successive time interval (2-7) show a continuous decreasing trend from 0.80 to 0.52. Additional file 2 also shows the results of principal components analyses of the correlation matrices. The first two components only are shown since they capture over 80% of the total covariation. For each of the three traits, the first component has loadings that all are positive in sign and similar in magnitude for the 7 intervals. For distance and duration, the second component primarily contrasts values for the first 3 intervals with those for the last 3 intervals (with interval 4 being a transition time). For speed, this component contrasts the first two intervals with the last four intervals (interval 3 is a transition time). For all three traits, therefore, this suggests that there is a change in the covariation structure of the values in the early versus late time intervals.

### Activity trait QTLs

The results of the genome scan for QTLs affecting the activity traits in each of the seven intervals are given in Additional files 3 (distance), 4 (duration), and 5 (speed). We designated these QTLs *DIST*, *DUR*, and *SPD *followed by their appropriate chromosome number and an extension to indicate whether they were the first or second QTL on each chromosome. Chromosomewise threshold LPR values generated from the permutation procedure varied from 1.82 to 2.47, averaging 2.08. Genomewise threshold LPR values varied in a narrow range from 3.33 to 3.58.

For distance (Additional file 3), a total of 13 QTLs on 9 different chromosomes reached statistical significance, including two on chromosomes 8, 9, 10, and 13. In addition, two QTLs were sex-specific, one on chromosome 7 affecting females only and one on chromosome 9 affecting only males. Only one QTL, *DIST9.1*, affected all 7 distance traits, the LPR scores for 5 of which reached significance at the genomewise level. Three other QTLs, *DIST5.1*, *DIST8.1 *and *DIST13.1*, affected several different traits; so overall, 4 of the 13 QTLs exhibited pleiotropy.

The distance QTLs primarily exhibit significant additive genotypic effects, their absolute values averaging 0.26 standard deviations. Most (23 of 30) of the *a *values also are positive in sign, suggesting that the C57L/J allele at these loci tends to increase the distance run more so than the C3H/HeJ allele. Fewer (13/30) of the dominance genotypic values reach significance, but the average of their absolute values is 0.24 and many exhibit overdominance (*d *greater than *a*) or underdominance (*d *less than *a*), so dominance appears to be important for these QTLs. The trends of the additive and dominance genotypic values across the intervals for the four pleiotropic QTLs are generally consistent. *DIST5.1 *and *DIST13.1*, for example, exhibit primarily additive effects on the distance traits since all *a *values are positive and significant whereas none of the *d *values reach significance. *DIST9.1 *exhibits an interesting alternate pattern in which all additive effects are significant, but dominance genotypic values are significant only for early distance (first four intervals), declining to non-significance for late distance (last three intervals). The contribution of each QTL to the total phenotypic variability in the distance traits ranged from 3.2% to 11.1%, averaging 5.1%.

For duration (Additional file 4), again a total of 13 QTLs reached significance, including two on chromosomes 8 and 15. Three QTLs (chromosomes 4, 8, and 14) affect duration traits only in females. Seven of the 13 QTLs exhibit pleiotropy, although mostly affect duration only in the early (1-3) or late (4-7) time intervals. Thus *DUR4.1*_*F*_, *DUR9.1*, and *DUR14.1*_*F *_affect only early duration whereas *DUR5.1*, *DUR6.1*, and *DUR13.1 *affect late duration. The duration QTLs exhibit nearly the same frequency of significant additive (14/23) and dominance genotypic effects (12/23). Dominance effects appear to be more important since the mean of the absolute *d *values (0.32) well exceeds that for the *a *values (0.22). This disparity partly has been generated because of the particularly high *d *values exhibited by all three female-specific QTLs. Negative *d *values outnumber positive *d *values, and a number of QTLs exhibit significant underdominance. One noteworthy trend is that all three pleiotropic QTLs previously identified as affecting early duration traits show nonsignificant additive but significant dominance effects whereas the reverse is true for the late duration traits (with one exception, the effect of *DUR5.1 *on DR7). The contribution of each QTL to the total phenotypic variability in the duration traits averaged 5.5%.

For speed (Additional file 5), again we discovered a total of 13 QTLs that reached significance, including two on chromosomes 2, 5, 8, and 13. One QTL on chromosome 11 affected males only and QTLs on chromosome 15 and 19 affected females only. Six of these QTLs showed pleiotropic effects, including one on chromosome 9 that affected all 7 speed traits. Among these pleiotropic QTLs, *SPD5.1 *affects only early speed whereas *SPD5.2*, *SP8.2*, and *SPD13.1 *affect only late speed. Twenty-five of the 31 total additive effects generated by the speed QTLs are positive in sign, again implying that the C57L/J (fast strain) allele tended to increase speed more so than the C3H/HeJ allele (slow strain). All but four of these *a *values also reached statistical significance whereas about half (15) of the *d *values were significant. However, means of the absolute *a *(0.28) and *d *values (0.27) were nearly identical, suggesting an important role for dominance. As was true for *DIST9.1*, *SPD9.1 *also exhibits significant dominance effects for early (SP1-SP3) but not late (SP4-SP7) speed. However, this trend does not always hold for the other pleiotropic QTLs; for example, *SPD8.1 *shows dominance for both early and late speed, and SPD5.1 effects on early speed are additive only. The contribution of each QTL to the total phenotypic variability in the speed traits averaged 5.5%.

**Figure 1:**
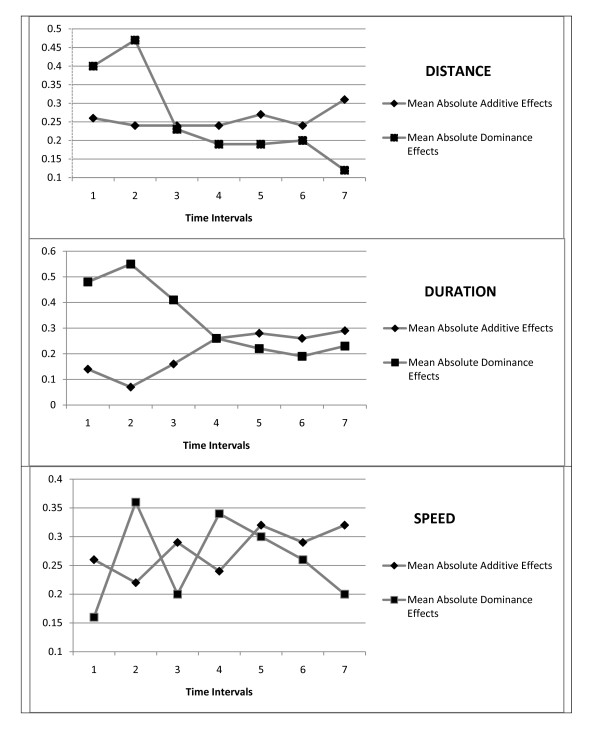
**Trends in the QTL effects on the activity traits over the seven intervals**. Shown are the means of the absolute additive and dominance genotypic values for the QTLs affecting distance, duration, and speed throughout the intervals.

Additional file 6 summarizes the numbers and effects of the QTLs discovered for the activity traits throughout the seven time intervals and Figure 1 illustrates these trends. The numbers of QTLs vary from 2 to 6 over time, with no discernable trend in the early or late ages. As a consequence of the relatively low numbers of QTLs affecting these traits, their percentage impact on the total phenotypic variability also is generally low, varying only from 6.4% to 27.9%. The higher percentages tend to be associated with greater numbers of significant QTLs since the average effect of these QTL varies only from about 2.4% to 5.6%. The average of the absolute additive genotypic values tends to increase in magnitude over the 7 intervals, although this trend is significant (*b *= +0.034, *P *= 0.012) only for duration. The average of the absolute dominance genotypic values tends to decrease throughout the age groups for distance (*b *= -0.051, *P *= 0.013) and duration (*b *= -0.059, *P *= 0.007), although not for speed (*b *= 0.001, *P *= 0.97). For distance and duration especially, therefore, dominance is more important in the early ages and tends to decrease in the later age intervals.

In the genetic analysis of regression slopes, we discovered only 3 QTLs for distance, 3 for duration, and 1 for speed (Additional file 7). Two of the QTLs for both distance and duration are located on chromosome 9 at locations (20 and 84 cM) that differ from those for the QTLs (*DIST9.1*, *DIST9.2*, *DUR9.1*, *DUR9.2*) previously found for the interval traits (Additional files 3, 4). The QTL at 84 cM reaches genomewise significance for both traits, and accounts for nearly 11% (distance) and 8% (duration) of the total phenotypic variation in the slopes of these traits over time. The only QTL discovered for speed slopes also is at a very similar location, suggesting that there is a single QTL on the distal part of chromosome 9 that strongly influences the trends in all three activity traits over this 21-day period. The remaining QTLs affecting distance and duration are female-specific, and that for distance on chromosome 19 again is independent from any others found. The location (and pleiotropy test) of the QTL for duration slopes (*DURB4.1*_*F*_), however, suggests it probably is the same as a female-specific QTL for duration previously found (*DUR4.1*_*F*_). All 7 QTLs exhibit a great deal of dominance. In fact, all of the dominance genotypic values exceed the additive genotypic values, and 5 of the 7 individual *d *values reach significance.

**Figure 2:**
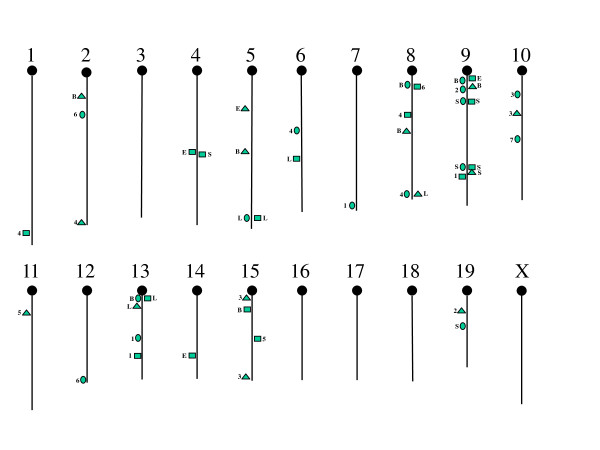
**All QTLs affecting the activity traits**. Shown are the chromosomal locations for the QTLs affecting distance (ovals), duration (rectangles), and speed (triangles) in two or more early intervals (E = 1-3) only, in two or more late intervals (L = 4-7) only, in both early and late intervals (B), or across all intervals (slopes = S).

Figure 2 provides an illustration of the locations of all 39 QTLs discovered for the activity traits in the various intervals, and indicates whether these QTLs affected traits only in the early (E = 1-3) or late (L = 4-7) intervals, in both intervals (B), or only in one interval (numbers). As may be seen, there is some colocalization of QTLs affecting distance (ovals), duration (rectangles), and speed (triangles) that is especially noticeable on chromosome 8, 9, and 13. The 7 QTLs found for the slopes of these traits over all intervals generally are separate from those affecting the activity traits in specific intervals.

## Discussion

The basic objective of this study was to better understand the genetics of voluntary physical activity within individuals. For this purpose, we averaged the distance, duration, and speed run each day by the F_2 _mice over seven three-day intervals, and searched for QTLs affecting these traits in each time interval. We were successful in discovering 13 different QTLs affecting each of the three traits (39 total QTLs) in one or more intervals. We assume that most of these are true QTLs because as previously explained, only about one false positive result is expected using the chromosomewise levels of significance.

The number of activity QTLs we discovered is more than double the number of total QTLs (18) originally found affecting the three activity traits obtained by averaging daily values over the entire three-week period in mice from this same population [12]. These results are not particularly surprising given that we analyzed more (21) traits than the three used in the previous study [12]. They do suggest, however, that the genetic architecture of wheel-running activity traits in mice is more complex than previously realized and can vary considerably depending on the specific time interval chosen for measurement.

### Concordance of QTLs

Among the 39 time-specific QTLs we discovered, 15 appear to be the same as those previously shown by Lightfoot *et al*. [12] to significantly affect the activity traits over the entire 21-day period. Only three QTLs found by Lightfoot *et al*. [12], including QTLs on chromosome 13 affecting distance and duration and one QTL on chromosome 12 affecting speed, were not detected in our genome scan. Not surprisingly, nearly all (13) of the matching 15 QTLs that we discovered exhibited pleiotropic effects on two or more activity traits. These included all those QTLs previously identified as affecting three or more traits (see Additional files 3, 4, 5). In general, therefore, QTLs capable of significantly influencing the activity traits over an extended 21-day period also tended to affect the traits over several different time intervals as well. There were six other QTLs not previously found that exhibited pleiotropy as well, including five affecting duration and one affecting speed, but each of these QTLs affected traits only over two time intervals.

We also discovered a number of other QTLs that affected the activity traits in just one age interval. Most of these QTLs generally were not detected in the previous study [12] that used the average activity over the entire 21-day period, and generally expand the genomic regions potentially involved in the control of physical activity. In fact, we found QTLs affecting one or more of the activity traits on all chromosomes except 3 (7 SNPs coverage) 16, 17, 18 (each with 5 SNPs coverage) and X (9 SNPs coverage). While all of the QTLs affecting traits in a single time interval reached significance only at the chromosomewise level and so need verification in future studies, they suggest that the majority of chromosomes may harbor genes involved in the control of physical activity, as might be expected for a moderately heritable complex trait controlled by many genes with relatively small effects. Our results are applicable only to the specific F_2 _population we generated from crossing the C57L/J and C3H/HeJ strains, and as QTL studies are performed with other progenitor strains, we should expect additional QTLs to be found, including on the four chromosomes where we found none.

### QTLs affecting early versus late activity

It was interesting that there was a tendency of the pleiotropic QTLs for duration to have an effect primarily either in the early or the late age intervals. This occurred in six of seven possible instances, only *DUR15.1 *exhibiting pleiotropic effects on duration in both early (DR3) and late intervals (DR7). It is possible that this result may simply be a consequence of limited statistical power to detect QTLs influencing duration throughout both the early and late intervals. To the extent that this is true, it would suggest caution in postulating the existence of separate sets of QTLs that affect duration only at certain ages. However, inspection of the highest LPR scores among all seven intervals for the six chromosomes on which the QTLs affecting only early or late duration are located reveals a rather clear and consistent trend. As one example, *DUR5.1 *significantly affected duration during the late (4-7) intervals only (Additional file 4), and the highest LPR scores on chromosome 5 for intervals 1 through 3, respectively, were 1.15, 1.18, and 1.74. This suggests that there is a gene on chromosome 5 that becomes increasingly active in the later intervals rather than simply being undetectable in the early intervals because of insufficient statistical power.

It is probable that the intra-individual genetic architecture of duration is fundamentally different from the other two activity traits. It is suggestive in this regard that there was a QTL on chromosome 9 that affected both distance (*DIST9.1*) and speed (*SPD9.1*) throughout all 7 time intervals, but the comparable QTL for duration at this location (*DUR9.1*) was significant only for two early age intervals (see Additional file 4). In the only other mouse study we are aware of that examined intraspecific genetic trends in wheel-running activity traits, Kelly *et al*. [19] found one QTL on chromosome 7 that affected duration (time) on five of the six days tested, but the other six QTLs discovered did not exhibit pleiotropy, each affecting duration only on a single day. Other pleiotropic QTLs were found on chromosomes 1 and 6 that affected the distance run by the mice only during the first two days of exposure to the running wheels [19], but these most likely represent behavior genes since this was their first exposure to the wheels.

Although it is not yet clear whether we should generally expect to find separate QTLs affecting physical activity at early or at late ages, such a trend has been seen for other complex traits. For example, Cheverud *et al*. [29] measured body weight each week for 10 weeks in over 500 F_2 _mice, and discovered two distinct sets of QTLs affecting growth that generally mapped to different genomic positions. One set of QTLs primarily affected growth in early (weeks 1-3) to middle (weeks 4-6) periods while another set affected middle to late growth (weeks 7-10), with very few QTLs affecting the entire postnatal growth period [29]. Rocha *et al*. [30] and Allan *et al*. [31] discovered the same sort of trend in their analysis of growth and body composition traits in mice. They also identified several QTL demonstrating differential regulation of regional adipose deposition and age-dependent regulation of growth and energy consumption. It is interesting that we found such a parallel result, at least for duration, even with a relatively short testing interval (3 weeks).

This pattern of effects of the pleiotropic QTLs is consistent with the major trend seen in the correlations among the activity traits in which they tended to decrease in magnitude between increasingly distant time intervals (Additional file 2). Thus the genetic contribution to the phenotypic covariation of traits arises primarily through pleiotropy [32], and we found few QTLs with pleiotropic effects on the activity traits that extended throughout most or all of the ages. This pleiotropic pattern also presumably is reflected in the contrast between early and late ages seen in the second component generated from the principal component analyses of the correlations of the activity traits. It should be noted that this sort of contrast could also be produced by QTLs exhibiting antagonistic pleiotropy, affecting the early traits in one direction and late traits in the opposite direction [33]. As we previously have seen (Additional files 3, 4, 5), however, the few QTLs we discovered that affected the activity traits throughout both early and late age intervals showed a consistent direction in their effects.

### Effects of activity QTLs

The majority of QTLs we discovered exhibited significant additive effects on the activity traits, especially for distance and speed. The QTLs affecting these two traits also were generally positive, signaling that alleles from the fast strain, C57L/J, generally tended to increase both the distance run as well as the speed. For duration, however, the numbers of positive and negative *a *values were nearly equal, and if only the significant *a *values are considered, the negative ones predominated (Additional file 4). This suggests that although the fast strain alleles generally acted to increase the distance the mice run, they also tended to decrease the duration of the run. This was not the case in the QTL study by Kelly *et al*. [19], however, where the distribution of positive and negative additive genotypic values was approximately the same for all three activity traits.

The QTLs we discovered often exhibited significant dominance genotypic values that averaged nearly the same (distance and speed) or even higher than the additive genotypic values (duration). This was as expected because in our previous analysis of 21-day activity traits, we found considerable dominance for the QTLs we discovered [12]. In addition, the F_1 _mice produced from the original cross of the C57L/6 and C3H/HeJ progenitors also exhibited heterosis since their mean exceeded that of the mean of the two parental strains for all three traits [12], and heterosis depends upon directional dominance [32]. Nehrenberg *et al*. [34] also observed significant heterosis for wheel-running behavior of mice in the F_1 _generation produced from the same two strains used as progenitors for the F_2 _generation of mice used by Kelly *et al*. [19] who noted the importance of dominance in the QTLs they discovered for physical activity. It therefore appears that dominance is an important component of the genetic architecture of wheel-running activity traits in mice.

The most interesting aspect about the dominance we observed was its prevalence especially in the QTLs for early compared with later activity. This pattern was most apparent for distance and duration rather than speed, and was sufficient to reach statistical significance in the regression analyses of the absolute dominance genotypic values for these two traits over the seven age intervals. Especially with the additive genotypic values generally increasing throughout the ages, this also means that the *d*/*a *ratio declined with age. This was particularly noticeable for duration, where the mean *d*/*a *ratio for the first three age intervals of 6.65 was much higher than that of 0.88 for the last three age intervals. Cheverud *et al*. [29] found a similar *d*/*a *trend exhibited by QTLs for body weight over a 10-week interval in their mouse population, and suggested that this strong dominance and/or overdominance for early growth may have arisen as a response to selection for an increased early growth rate.

### Nature of the activity QTLs

At present we have little information about the genes underlying these QTLs for the activity traits. Partly this is because of the imprecision of the mapping process in an F_2 _generation such as ours where the support intervals for the QTLs tend to be rather large. In addition, at present there are few studies that have searched for QTLs affecting activity traits in mice, so we have little data to use for comparison. And even where other studies may be available, they typically involve other inbred strain progenitors which are expected to yield QTLs uniquely polymorphic between those strains. A case in point is the mouse study by Kelly *et al*. [19] who discovered significant QTLs for distance and duration on chromosomes 1, 5, 6, and 7, and 19 (duration only) and for speed on chromosomes 2 and 17. The significant QTLs we found for the activity traits were on chromosomes 2 (speed only), 5, 8, 9, and 13. Thus the only chromosomes in common are 5 for distance and duration and 2 for speed, but even in these cases, the locations of the QTLs we discovered on these chromosomes do not overlap with those calculated by Kelly *et al*. [19].

It might be particularly useful to know the identity of the genes underlying the QTLs we discovered for the slopes of the activity traits. These QTLs were most interesting because, as was also found by Kelly *et al*. [19], their locations generally were different from those for the QTLs controlling activity in the individual intervals. This suggests that there is at least some genetic control of the trajectories of overall activity exhibited by our F_2 _mice that is different from that for activity in individual, early, or late time intervals. Should the genes underlying these QTLs eventually be identified, it is possible that they play an important role in the maintenance of physical activity over long periods of time.

## Conclusions

We have located a number of QTLs that influence the direction, duration, and speed run by mice in our F_2 _population over each of seven three-day intervals. Although some of these QTLs are apparently the same as found in our original study using the average of these physical activity traits measured daily over the entire 21-day period [12], a number are novel and affect these traits only over the early or the late age periods. We also identified several QTLs that affect the overall trajectories of activity during the entire testing period that appear to be independent from those affecting the activity traits in individual intervals. Although the nature of the genes underlying all of these QTLs remains unknown, these findings suggest that the genetic architecture of physical activity in our population of mice varies over time.

## Authors' contributions

LJL performed the data analysis, wrote and prepared the manuscript for submission. JTL was the principal supervisor of the study and assisted with preparation of the manuscript. DP designed the genome scan including SNP selection, and reviewed the manuscript. All authors read and approved the final manuscript.

## Supplementary Material

Additional file 1 **Basic statistics for the activity traits**. Shown are the means and standard deviations (Std) of distance (km/day), duration (min/day) and speed (meters/min) during each of the seven time intervals (1-7). The sample size = 310 in all cases. Click here for file

Additional file 2 **Correlations and principal components analyses for the activity traits**. Correlations are given for distance, duration, and speed traits for each of the 7 time intervals as well as loadings on the first two principal components, I and II, derived from a component analysis of the correlation matrix for each of the three traits. Click here for file

Additional file 3 **QTLs for distance**. Shown are the locations, confidence intervals (CI), LPR scores (log_10_Prob^-1^), percentage of the total phenotypic variation explained (%), and standardized additive (*a*) and dominance genotypic values (*d*) for QTLs on all chromosomes affecting distance in any of the 7 time intervals (DT1 through DT7). Each QTL for distance is designated *DIST *followed by its chromosome number and an extension to indicate whether it is the first or second QTL on that chromosome. Subscripts are given for QTLs if they affect only males (*M*) or only females (*F*). Locations are given as map distances from the nearest proximal marker (Marker Dist) and from the centromere (Cent. Dist) and confidence intervals are expressed as distances from the centromere. Single locations and confidence intervals are indicated for multiple traits when tests suggested pleiotropy of common QTLs. All LPR values are significant at the 5% chromosomewise level or at the genomewise level (†). * = *P *< 0.05; ** = *P *< 0.01. Click here for file

Additional file 4 **QTLs for duration**. Shown are the locations, confidence intervals (CI), LPR scores (log_10_Prob^-1^), percentage of the total phenotypic variation explained (%), and standardized additive (*a*) and dominance genotypic values (*d*) for QTLs on all chromosomes affecting duration in any of the 7 time intervals (DR1 through DR7). Each QTL is designated *DUR *followed by its chromosome number and an extension to indicate whether it is the first or second QTL on that chromosome. Subscripts are given for QTLs if they affect only males (*M*) or only females (*F*). Locations are given as map distances from the nearest proximal marker (Marker Dist) and from the centromere (Cent. Dist) and confidence intervals are expressed as distances from the centromere. Single locations and confidence intervals are indicated for multiple traits when tests suggested pleiotropy of common QTLs. All LPR values are significant at the 5% chromosomewise level or at the genomewise level (†). * = *P *< 0.05; ** = *P *< 0.01. Click here for file

Additional file 5 **QTLs for speed**. Shown are the locations, confidence intervals (CI), LPR scores (log_10_Prob^-1^), percentage of the total phenotypic variation explained (%), and standardized additive (*a*) and dominance genotypic values (*d*) for QTLs on all chromosomes affecting speed in any of the 7 time intervals (SP1 through SP7). Each QTL is designated SPD followed by its chromosome number and an extension to indicate whether it is the first or second QTL on that chromosome. Subscripts are given for QTLs if they affect only males (*M*) or only females (*F*). Locations are given as map distances from the nearest proximal marker (Marker Dist) and from the centromere (Cent. Dist) and confidence intervals are expressed as distances from the centromere. Single locations and confidence intervals are indicated for multiple traits when tests suggested pleiotropy of common QTLs. All LPR values are significant at the 5% chromosomewise level or at the genomewise level (†). * = *P *< 0.05; ** = *P *< 0.01. Click here for file

Additional file 6 **QTL summary statistics for the activity traits over the seven time intervals**. Shown are the number of QTLs affecting the activity traits over each of the seven time intervals, the percentage of the total phenotypic variation they contribute (expressed as a total and per QTL), and the means of their absolute additive (*a*) and dominance genotypic values (*d*). Click here for file

Additional file 7 **QTLs for the slopes of the activity traits over all seven intervals**. Shown are the locations, confidence intervals (CI), LPR scores (log_10_Prob^-1^), percentage of the total phenotypic variation explained (%), and standardized additive (*a*) and dominance genotypic values (*d*) for QTLs on all chromosomes affecting the regression (slope) of distance, duration, and speed over all intervals. Each QTL for distance is designated *DISTB, DURB, or SPDB *(for the slopes of distance, duration, and speed) followed by its chromosome number and an extension to indicate whether it is the first or second QTL on that chromosome. Subscripts are given for QTLs if they affect only males (*M*) or only females (*F*). Locations are given as map distances from the nearest proximal marker (Marker Dist) and from the centromere (Cent. Dist) and confidence intervals are expressed as distances from the centromere. Single locations and confidence intervals are indicated for multiple traits when tests suggested pleiotropy of common QTLs. All LPR values are significant at the 5% chromosomewise level or at the genomewise level (†). * = *P *< 0.05; ** = *P *< 0.01. Click here for file
