# Supplementary material for: A search for quantitative trait loci controlling within-individual variation of physical activity traits in mice
Source: BMC Genet. 2010 Sep 21;11:83. doi: 10.1186/1471-2156-11-83 (PMC2949740; doi:10.1186/1471-2156-11-83)
Supplement: Additional file 1 — Basic statistics for the activity traits. Shown are the means and standard deviations (Std) of distance (km/day), duration (min/day) and speed (meters/min) during each of the seven time intervals (1-7). The sample size = 310 in all cases. [file 1471-2156-11-83-S1.PDF]

**Additional file 1. Basic statistics for the activity traits**

| Trait | Statistic | 1 | 2 | 3 | 4 | 5 | 6 | 7 |
|---|---|---|---|---|---|---|---|---|
| Distance | Mean | 5.76 | 6.06 | 6.50 | 6.46 | 6.31 | 6.45 | 6.45 |
| | Std | 2.58 | 2.82 | 2.61 | 2.68 | 2.67 | 2.54 | 2.70 |
| Duration | Mean | 311.42 | 324.25 | 339.74 | 336.75 | 325.92 | 333.93 | 326.38 |
| | Std | 116.02 | 126.97 | 112.16 | 116.22 | 113.54 | 105.40 | 110.65 |
| Speed | Mean | 17.39 | 17.49 | 18.28 | 18.26 | 18.17 | 18.63 | 19.00 |
| | Std | 4.30 | 4.51 | 4.00 | 4.16 | 4.38 | 3.78 | 4.28 |

Shown are the means and standard deviations (Std) of distance (km/day), duration (min/day) and speed (meters/min) during each of the seven time intervals (1-7). The sample size = 310 in all cases.
