# Supplementary material for: A search for quantitative trait loci controlling within-individual variation of physical activity traits in mice
Source: BMC Genet. 2010 Sep 21;11:83. doi: 10.1186/1471-2156-11-83 (PMC2949740; doi:10.1186/1471-2156-11-83)
Supplement: Additional file 2 — Correlations and principal components analyses for the activity traits. Correlations are given for distance, duration, and speed traits for each of the 7 time intervals as well as loadings on the first two principal components, I and II, derived from a component analysis of the correlation matrix for each of the three traits. [file 1471-2156-11-83-S2.PDF]

Additional file 2. Correlations and principal components analyses for the activity traits

	I	II	2	3	4	5	6	7
				Distance				
1	0..37	-0.46	0.80	0.74	0.71	0.66	0.59	0.52
2	0.37	-0.46		0.80	0.69	0.66	0.60	0.53
3	0.39	-0.22			0.80	0.72	0.65	0.66
4	0.39	-0.03				0.80	0.72	0.65
5	0.39	0.21					0.80	0.70
6	0.38	0.45						0.79
7	0.35	0.53						
				Duration				
1	0.37	-0.43	0.76	0.75	0.70	0.65	0.57	0.47
2	0.37	-0.47		0.78	0.64	0.64	0.54	0.47
3	0.40	-0.20			0.78	0.74	0.65	0.63
4	0.39	-0.02				0.78	0.69	0.59
5	0.37	0.14					0.76	0.64
6	0.39	0.45						0.75
7	0.35	0.57						
				Speed				
1	0.37	-0.52	0.76	0.73	0.67	0.57	0.63	0.56
2	0.37	-0.55		0.77	0.63	0.56	0.64	0.56
3	0.41	-0.11			0.78	0.70	0.73	0.69
4	0.39	0.11				0.73	0.71	0.61
5	0.37	0.41					0.73	0.61
6	0.39	0.30						0.71
7	0.35	0.38						

Correlations are given for distance, duration, and speed traits for each of the 7 time intervals as well as loadings on the first two principal components, I and II derived from a component analysis of the correlation matrix for each of the three traits.
