# Supplementary material for: A search for quantitative trait loci controlling within-individual variation of physical activity traits in mice
Source: BMC Genet. 2010 Sep 21;11:83. doi: 10.1186/1471-2156-11-83 (PMC2949740; doi:10.1186/1471-2156-11-83)
Supplement: Additional file 3 — QTLs for distance. Shown are the locations, confidence intervals (CI), LPR scores (log10Prob-1), percentage of the total phenotypic variation explained (%), and standardized additive (a) and dominance genotypic values (d) for QTLs on all chromosomes affecting distance in any of the 7 time intervals (DT1 through DT7). Each QTL for distance is designated DIST followed by its chromosome number and an extension to indicate whether it is the first or second QTL on that chromosome. Subscripts are given for QTLs if they affect only males (M) or only females (F). Locations are given as map distances from the nearest proximal marker (Marker Dist) and from the centromere (Cent. Dist) and confidence intervals are expressed as distances from the centromere. Single locations and confidence intervals are indicated for multiple traits when tests suggested pleiotropy of common QTLs. All LPR values are significant at the 5% chromosomewise level or at the genomewise level (†). * = P < 0.05; ** = P < 0.01. [file 1471-2156-11-83-S3.PDF]

Additional file 3. QTLs for distance

QTL	Marker	Marker Dist (cM)	Cent. Dist (cM)	CI (cM)	DT Interval	LPR	*%*	*a/SD*	*d/SD*
DIST2.1	*rs13476352*	16	27	15 to 70	DT6	2.42	3.76	-0.26**	-0.21
DIST5.1	*rs13478553*	0	95	88 to 100	DT4	2.46	3.77	-0.29**	-0.04
					DT5	2.84	4.38	-0.32**	-0.05
					DT6	2.49	3.81	-0.29**	0.01
					DT7	3.41	5.18	-0.32**	-0.17
DIST6.1	*gnf06.086.089*	0	47	21 to 84	DT4	2.48	3.91	0.27**	0.11
DIST7.1$_F$	*rs3714636*	0	94	90 to 96	DT1	2.60	8.20	0.25	0.60**
DIST8.1	*rs13479600*	8	10	2 to 30	DT2	2.20	3.27	0.17*	-0.32*
					DT3	2.14	3.16	0.25**	-0.05
					DT4	2.81	4.44	0.22*	-0.34*
					DT5	2.68	3.97	0.14	-0.40**
					DT6	3.28	4.83	0.18*	-0.41**
DIST8.2	*rs3703161*	0	84	78 to 84	DT4	2.12	3.32	-0.09	0.36**
DIST9.1	*rs13480073*	4	7	1 to 27	DT1	4.26†	6.55	0.21*	0.50**
					DT2	4.26†	6.55	0.21*	0.48**
					DT3	5.22†	7.96	0.22**	0.52**
					DT4	4.09†	6.29	0.29**	0.26*
					DT5	3.20	4.95	0.26**	0.19
					DT6	3.61†	5.58	0.28**	0.21
					DT7	3.07	4.76	0.28**	0.15
DIST9.2$_M$	*rs13480273*	10	55	50 to 65	DT2	3.67	11.14	0.34**	-0.60**
DIST10.1	*rs3712998*	12	14	1 to 38	DT3	2.26	3.50	0.25**	0.11
DIST10.2	*rs13480273*	4	50	15 to 84	DT7	2.35	3.71	0.29**	0.04

DIST12.1	*rs6361467*	0	68	44 to 68	DT6	2.07	3.19	0.10	-0.31**
DIST13.1	*rs6329684*	0	1	1 to 5	DT1	3.33	5.18	0.31**	0.16
					DT4	2.40	3.72	0.27**	-0.01
					DT5	4.25†	6.51	0.34**	0.13
					DT6	4.28†	6.55	0.35**	-0.04
					DT7	3.91†	5.99	0.33**	0.10
DIST13.2	*rs13481855*	10	43	35 to 43	DT1	2.90	4.58	-0.27**	-0.33*

Shown are the locations, confidence intervals (CI), LPR scores ($\log_{10}\text{Prob}^{-1}$), percentage of the variation explained (%), and standardized additive (*a*) and dominance genotypic values (*d*) for QTLs on all chromosomes affecting distance in any of the 7 time intervals (DT1 through DT7) Each QTL for distance is designated *DIST* followed by its chromosome number and an extension to indicate whether it is the first or second QTL on that chromosome. Subscripts are given for QTLs if they affect only males (*M*) or only females (*F*). Locations are given as map distances from the nearest proximal marker (Marker Dist) and from the centromere (Cent. Dist) and confidence intervals are expressed as distances from the centromere. Single locations and confidence intervals are indicated for multiple traits when tests suggested pleiotropy of common QTLs. All LPR values are significant at the 5% chromosomewise level or at the genomewise level (†). * = $P < 0.05$; ** = $P < 0.01$.
