# Supplementary material for: A search for quantitative trait loci controlling within-individual variation of physical activity traits in mice
Source: BMC Genet. 2010 Sep 21;11:83. doi: 10.1186/1471-2156-11-83 (PMC2949740; doi:10.1186/1471-2156-11-83)
Supplement: Additional file 6 — QTL summary statistics for the activity traits over the seven time intervals. Shown are the number of QTLs affecting the activity traits over each of the seven time intervals, the percentage of the total phenotypic variation they contribute (expressed as a total and per QTL), and the means of their absolute additive (a) and dominance genotypic values (d). [file 1471-2156-11-83-S6.PDF]

Additional file 6. QTL summary statistics for the activity traits over the seven time intervals

Time Intervals		1	2	3	4	5	6	7
Distance	No. QTLs	4	3	3	6	4	6	4
	% Var.	14.5	12.5	14.2	20.6	17.6	22.9	17.2
	%/QTLs	3.6	4.2	4.7	3.4	4.4	3.8	4.3
	Mean |a|	0.26	0.24	0.24	0.24	0.27	0.24	0.31
	Mean |d|	0.40	0.47	0.23	0.19	0.19	0.20	0.12
Duration	No. QTLs	3	2	4	4	4	3	3
	% Var.	10.1	6.4	9.7	13.8	14.5	9.6	12.7
	%/QTLs	3.4	3.2	2.4	3.5	3.6	3.2	4.2
	Mean |a|	0.14	0.07	0.16	0.26	0.28	0.26	0.29
	Mean |d|	0.48	0.55	0.41	0.26	0.22	0.19	0.23
Speed	No. QTLs	3	5	6	4	5	5	3
	% Var.	13.7	19.3	24.7	12.2	15.9	27.9	18.2
	%/QTLs	4.5	3.9	4.1	3.5	3.2	5.6	6.1
	Mean |a|	0.26	0.22	0.29	0.24	0.32	0.29	0.32
	Mean |d|	0.16	0.36	0.20	0.34	0.30	0.26	0.20

Shown are the number of QTLs affecting the activity traits over each of the seven time intervals, the percentage of variation they contribute (expressed as a total and per QTL), and the means of their absolute additive (*a*) and dominance genotypic values (*d*).
