# Supplementary material for: A search for quantitative trait loci controlling within-individual variation of physical activity traits in mice
Source: BMC Genet. 2010 Sep 21;11:83. doi: 10.1186/1471-2156-11-83 (PMC2949740; doi:10.1186/1471-2156-11-83)
Supplement: Additional file 7 — QTLs for the slopes of the activity traits over all seven intervals. Shown are the locations, confidence intervals (CI), LPR scores (log10Prob-1), percentage of the total phenotypic variation explained (%), and standardized additive (a) and dominance genotypic values (d) for QTLs on all chromosomes affecting the regression (slope) of distance, duration, and speed over all intervals. Each QTL for distance is designated DISTB, DURB, or SPDB (for the slopes of distance, duration, and speed) followed by its chromosome number and an extension to indicate whether it is the first or second QTL on that chromosome. Subscripts are given for QTLs if they affect only males (M) or only females (F). Locations are given as map distances from the nearest proximal marker (Marker Dist) and from the centromere (Cent. Dist) and confidence intervals are expressed as distances from the centromere. Single locations and confidence intervals are indicated for multiple traits when tests suggested pleiotropy of common QTLs. All LPR values are significant at the 5% chromosomewise level or at the genomewise level (†). * = P < 0.05; ** = P < 0.01. [file 1471-2156-11-83-S7.PDF]

Additional file 7. QTLs for the slopes of the activity traits over all seven intervals.

QTL	Marker	Marker Dist (cM)	Cent. Dist (cM)	CI (cM)	Interval	LPR	*%*	*a/SD*	*d/SD*
DISTB9.1	*rs3713675*	4	20	7 to 36	All	2.25	4.10	0.14*	-0.36
DISTB9.2	*rs13480409*	8	84	78 to 89	All	5.21†	10.66	-0.21*	0.69
DISTB19.1$_F$	*rs6259521*	8	29	15 to 37	All	2.76	8.63	0.02	-0.79**
DURB4.1$_F$	*rs4136370*	2	61	55 to 71	All	2.32	7.89	0.01	0.70**
DURB9.1	*rs3713675*	4	20	5 to 34	All	2.04	3.27	0.04	-0.37**
DURB9.2	*rs13480409*	8	84	78 to 89	All	6.56†	9.89	-0.12	0.71**
SPDB9.1	*rs13480409*	12	88	80 to 91	All	3.44†	6.30	0.14*	0.36**

Shown are the locations, confidence intervals (CI), LPR scores ($\log_{10}\text{Prob}^{-1}$), percentage of the variation explained (%), and standardized additive (*a*) and dominance genotypic values (*d*) for QTLs on all chromosomes affecting the regression (slope) of distance, duration, and speed over all intervals. Each QTL for distance is designated *DISTB, DURB, or SPDB* (for the slopes of distance, duration, and speed) followed by its chromosome number and an extension to indicate whether it is the first or second QTL on that chromosome. Subscripts are given for QTLs if they affect only males (*M*) or only females (*F*). Locations are given as map distances from the nearest proximal marker (Marker Dist) and from the centromere (Cent. Dist) and confidence intervals are expressed as distances from the centromere. Single locations and confidence intervals are indicated for multiple traits when tests suggested pleiotropy of common QTLs. All LPR values are significant at the 5% chromosomewise level or at the genomewise level (†). * = $P < 0.05$; ** = $P < 0.01$.
